# Platelet-to-lymphocyte ratio: a predictive biomarker for exocrine gland involvement in Sjögren disease

**DOI:** 10.1186/s42358-025-00490-3

**Published:** 2025-11-11

**Authors:** Minchao Zou, Yitian Shi, Wei Xu, Yunxia Hu, Kejia Yu, Ting Liu, Fenghong Yuan

**Affiliations:** Department of Rheumatology and Immunology, The Affiliated Wuxi People’s Hospital of Nanjing Medical University, Wuxi People’s Hospital, Wuxi Medical Center, Nanjing Medical University, Wuxi, 214023 China

**Keywords:** Sjögren disease, Platelet-to-lymphocyte ratio, Tear meniscus height, Labial gland biopsy

## Abstract

**Objective:**

To investigate the relationship between the platelet-to-lymphocyte ratio (PLR) and ocular surface damage as well as labial gland pathology in patients with Sjögren disease (SjD).

**Methods:**

This retrospective study included 181 newly diagnosed SjD patients. Blood tests were used to calculate PLR, systemic immune-inflammation index (SII), neutrophil-to-lymphocyte ratio (NLR), and fibrinogen-to-albumin ratio (FAR). All patients underwent comprehensive ocular surface examinations and labial gland biopsies. Spearman correlation analysis was used to evaluate the relationships between variables, and receiver operating characteristic (ROC) curves determined optimal cutoff values for PLR in predicting tear meniscus height (TMH) abnormality and labial gland pathology positivity. Mann-Whitney U tests identified potential risk factors for labial gland pathology positivity. The significant factors (*P* < 0.05) from the univariate analysis were incorporated into a binary logistic regression model to determine independent risk factors.

**Results:**

(1) Correlation analysis showed that PLR was positively correlated with SII (*r* = 0.680, *P* < 0.001), NLR (*r* = 0.488, *P* < 0.001), FAR (*r* = 0.162, *P* = 0.031), and labial gland pathology (*r* = 0.159, *P* = 0.035), while PLR was negatively correlated with TMH (*r*=-0.202, *P* = 0.008). (2) ROC analysis revealed that PLR had an area under the curve (AUC) of 0.609 (95%CI: 0.522–0.695, *p* = 0.015) for predicting abnormal TMH, with a sensitivity of 48.6% and specificity of 74% at a cutoff of 151.34. For predicting labial gland pathology positivity, PLR had an AUC of 0.616 (95% CI: 0.503–0.730, *p* = 0.035), with a sensitivity of 65.7% and specificity of 58.8% at a cutoff of 122.92. (3) Binary logistic regression analysis showed that the high PLR group(PLR > 122.92) had 2.8 times the risk of labial gland pathology positivity compared to the low PLR group (OR = 2.842, 95%CI: 1.271–5.358, *p* = 0.011).

**Conclusion:**

PLR is a useful inflammatory marker for evaluating exocrine gland involvement in SjD patients.

## Introduction

Sjögren disease (SjD) is a chronic autoimmune disease characterized by prominent lymphoproliferative features and immune dysregulation, particularly B-cell overactivation. This drives autoantibody production, disease progression from organ-specific to systemic involvement, and an increased risk of B-cell lymphoma [1]. A key feature of SjD is lymphocytic infiltration of exocrine glands, especially the salivary and lacrimal glands, resulting in glandular dysfunction and classic symptoms such as dry eyes and mouth. Inflammation plays a central role in mediating tissue damage and disease progression.

Recently, hematological indicators reflecting systemic inflammation and disease severity have garnered increasing attention. Among emerging biomarkers, the platelet-to-lymphocyte ratio (PLR) stands out for its simplicity, cost-effectiveness, and clinical feasibility [2]. PLR is based on the dynamic relationship between two key components of the immune system: platelets, which are crucial for initiating and propagating inflammation, and lymphocytes, which are the primary mediators of immune responses. Although PLR has shown promise across various inflammatory and autoimmune diseases, its potential role in SjD remains inadequately explored. This study aims to evaluate the association between PLR and ocular surface damage as well as labial gland pathology in SjD, and further explore the clinical application potential of PLR.

## Patients and methods

### Subjects

This retrospective study enrolled 181 adult patients with SjD admitted to the Affiliated Wuxi People’s Hospital of Nanjing Medical University (Wuxi, Jiangsu, China) between April 2021 and March 2025. All SjD patients were newly diagnosed without treatment based on the 2016 American College of Rheumatology(ACR)/European League Against Rheumatism(EULAR) classification criteria [3]. Patients with active infections, malignancies, lymphoproliferative disorders, hematologic diseases, diabetes, other autoimmune diseases, or other ophthalmic conditions were excluded. Additionally, individuals with a history of contact lens use were also excluded. The research protocol was approved by the Institutional Research Ethics Committee of the Wuxi People’s Hospital (No. KS202035). The study was conducted in accordance with the Declaration of Helsinki. Written informed consent was obtained from all individual participants included in the study.

### Laboratory assessments

Laboratory evaluations included complete blood count (CBC), erythrocyte sedimentation rate (ESR), C-reactive protein (CRP), fibrinogen, albumin, globulin, immunoglobulin G (IgG), and serum autoantibodies. We calculated the platelet-to-lymphocyte ratio (PLR), systemic immune-inflammation index (SII), neutrophil-to-lymphocyte ratio (NLR), and fibrinogen-to-albumin ratio (FAR) as follows: PLR = platelet count/lymphocyte count; SII = platelet count × neutrophil count/lymphocyte count; NLR = neutrophil count/lymphocyte count; FAR = fibrinogen/albumin.

### Ocular surface examinations

All patients underwent comprehensive ocular surface examinations by an ocular surface analyzer (Oculus Company, Germany) to measure tear meniscus height (TMH), non-invasive keratography average break-up time (av-NIKBUT), and meibomian gland drop score (MGDS). MGDS was assessed using a subjective 4-grade scoring system for gland loss in both eyelids: 0 (no gland loss), 1 (≤ 33% gland loss), 2 (33%–67% gland loss), and 3 (> 67% gland loss) [4, 5]. Abnormal TMH was defined as < 0.2 mm.

### Labial gland biopsy

A standardized procedure was used to remove two to four glands from the lower labial mucosa under topical anesthesia. The glands were fixed in 4% formalin and embedded in paraffin. Sections were stained with hematoxylin–eosin (H&E). Histological evaluation was performed independently by two pathologists blinded to other test results, including ocular investigations and blood tests. A positive outcome was defined as a focus score (FS) ≥ 1 (FS: ≥50 lymphocytic cells per 4 mm²).

### Statistical analysis

Statistical analyses were performed using IBM SSJD Statistics 27 (IBM Corp., Armonk, NY, USA). Descriptive statistics are presented as counts, percentages, medians, and interquartile ranges. Normality of quantitative data was assessed using the Shapiro-Wilk test. The Pearson χ² test was used for categorical variables. Spearman correlation analysis was used to evaluate relationships between variables, with a correlation coefficient *r* ≥ 0.5 indicating moderate or stronger relationships. Receiver operating characteristic (ROC) curve analysis was used to determine the optimal cutoff values of PLR for predicting TMH abnormality and labial gland pathology positivity. Mann-Whitney U tests were used to identify potential risk factors associated with labial gland pathology positivity. The significant factors (*P* < 0.05) from the univariate analysis were incorporated into a binary logistic regression model to determine independent risk factors. *p* < 0.05 was considered statistically significant.

## Results

### Patient characteristics

The study included 181 patients with SjD. The median age was 52 (40.5–60.5) years, and 177 patients (97.8%) were female. The median disease duration was 12 (6–48) months. Table 1 shows the results of ocular surface examination. Labial gland pathology was positive in 81.2% of patients. The positive rates were respectively 74.0%, 72.4%, 43.1%, 99.4%, 24.3%, and 56.4% for anti-SSA/Ro52, anti-SSA/Ro60, anti-SSB/La, ANA(Anti-Nuclear Antibody), ACA(​Anti-Centromere Antibody), and RF(Rheumatoid Factor).

**Table 1:** Ocular surface examination results

	OD	OS
TMH (mm)	0.22(0.17,0.27)	0.21(0.16,0.27)
av-NIKBUT (s)	6.18(4.30,11.09)	6.99(4.38,11.18)
MGDS	1(1,2)	2(1,2)

Values are presented as median (interquartile range)

Units for ocular surface examinations: TMH (mm); av-NIKBUT (s)

Spearman correlation analysis was performed on ocular surface examination parameters (TMH, av-NIKBUT, and MGDS) of both eyes for all patients (Table 2). The results revealed moderate to high correlations between the right and left eyes for TMH (*r* = 0.716, *p* < 0.001), av-NIKBUT (*r* = 0.626, *p* < 0.001), and MGDS (*r* = 0.821, *p* < 0.001). Due to the high correlation between the parameters of both eyes(Table 2), only left eye (OS) data were used in the subsequent analysis.

**Table 2:** Spearman correlation analysis of bilateral TMH, av-NIKBUT and MGDS

TMH(OD)	--					
TMH(OS)	0.716^**^	--				
av-NIKBUT(OD)	0.213^**^	0.173^*^	--			
av-NIKBUT(OS)	0.152^*^	0.207^**^	0.626^**^	--		
MGDS(OD)	-0.061	-0.178^*^	0.018	-0.001	--	
MGDS(OS)	-0.066	-0.156^*^	-0.025	0.009	0.821^**^	--
	TMH(OD)	TMH(OS)	av-NIKBUT(OD)	av-NIKBUT(OS)	MGDS(OD)	MGDS(OS)

*P < 0.05, **P < 0.01

Table 3 shows the correlation analysis of PLR with age, immune-inflammatory indicators, ocular surface parameters, and labial gland pathology. Spearman correlation analysis revealed that PLR was positively correlated with SII (*r* = 0.680, *P* < 0.001), NLR (*r* = 0.488, *P* < 0.001), FAR (*r* = 0.162, *P* = 0.031), and labial gland pathology (*r* = 0.159, *P* = 0.035), and negatively correlated with TMH (*r* = -0.202, *P* = 0.008).

**Table 3:** Spearman correlation analysis of PLR with Age, Immune-Inflammatory Indicators, ocular surface Parameters, and labial gland pathology

	PLR	
	*r*	*P* Value
Age	-0.071	0.345
Disease duration	-0.109	0.150
CRP(C-reactive protein)	0.034	0.654
ESR(erythrocyte sedimentation rate‌)	0.118	0.117
SII	0.680	< 0.001*
NLR	0.488	< 0.001*
FAR	0.162	0.031*
G(globulin)	0.000	0.995
IgG(Immunoglobulin G)	-0.024	0.755
ANA	0.052	0.491
TMH	-0.202	0.008*
av-NIKBUT	0.089	0.241
MGDS	0.112	0.144
Labial gland pathology	0.159	0.035*

**Fig. 1:**
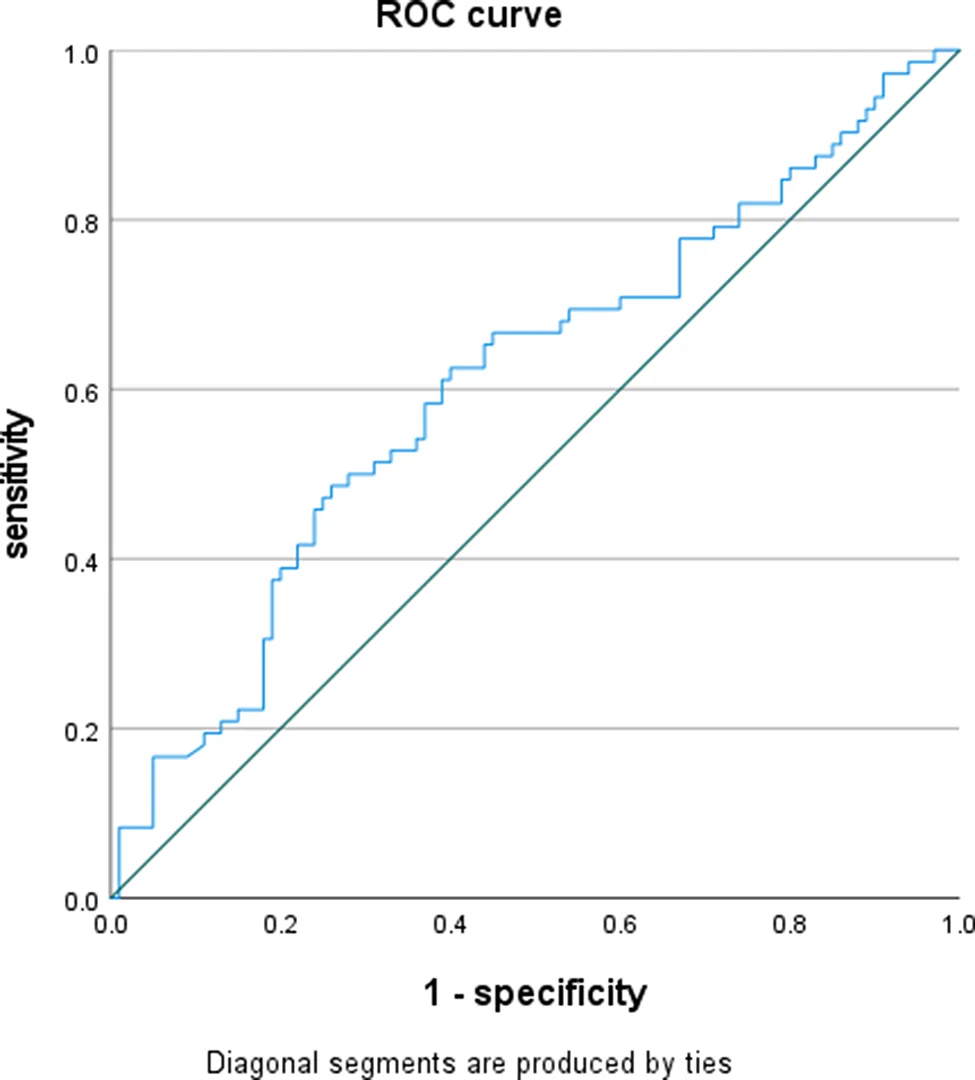
ROC curve of PLR for predicting TMH abnormality

**Fig. 2:**
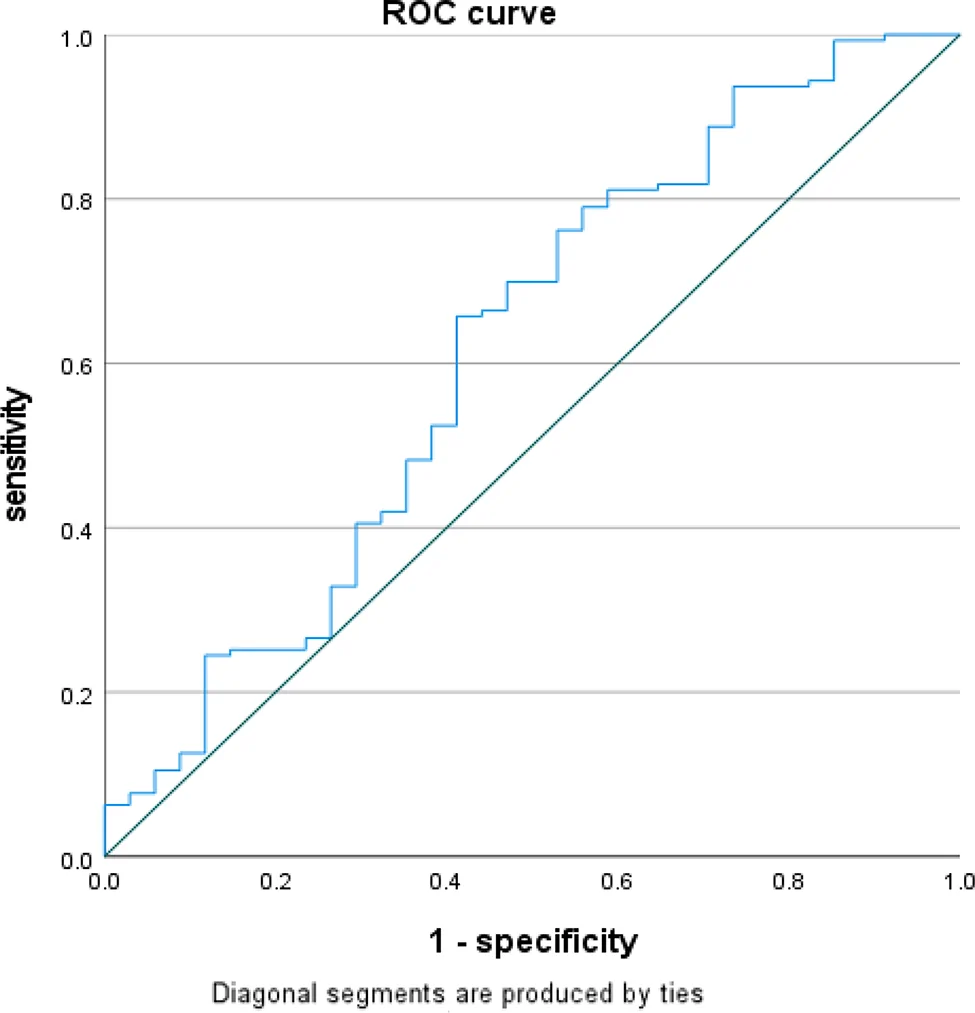
ROC curve of PLR for predicting labial gland pathology positivity

To further evaluate the clinical utility of PLR, ROC curve analyses were performed (Figs. 1 and 2). PLR showed an AUC of 0.609 (95% CI: 0.522–0.695, *P* = 0.015) for predicting TMH abnormality (< 0.2 mm), with a sensitivity of 48.6% and specificity of 74.0% at a cutoff of 151.34. For predicting labial gland pathology positivity, PLR had an AUC of 0.616 (95% CI: 0.503–0.730, *P* = 0.035), yielding 65.7% sensitivity and 58.8% specificity at a cutoff of 122.92.

To evaluate whether PLR is an independent risk factor for labial gland pathology, univariate analysis was first performed to identify indicators significantly associated with labial gland pathology (Table 4), which included ESR (z = -2.364, *P* = 0.018), PLR (z = -2.108, *P* = 0.035), ANA (z = -2.760, *P* = 0.006), and av-NIKBUT (z = -2.128, *P* = 0.033). These indicators were subsequently incorporated into a binary logistic regression model for multivariate analysis (Table 5). The analysis revealed that PLR was an independent predictor of labial gland pathology positivity (OR = 1.01, 95% CI: 1.001–1.019, *P* = 0.038) after adjusting for other variables. To further validate the predictive capacity of PLR, patients were stratified into high PLR (> 122.92) and low PLR (≤ 122.92) groups based on the optimal cutoff value derived from ROC curve analysis, and stratified analysis was conducted (Table 6). After controlling for the same confounding factors, the high PLR group demonstrated a 2.8-fold increased risk of labial gland pathology positivity compared to the low PLR group (OR = 2.842, 95% CI: 1.271–5.358, *P* = 0.011).

**Table 4:** Univariate analysis of factors associated with positive labial gland pathology

	Negative Labial Gland Pathology Group (*n* = 34)	Positive Labial Gland Pathology Group (*n* = 147)	x^2^/z	*P* Value
Age	52.5(44.5,60)	52(40,61.25)	-0.881	0.378
Disease duration	24(5.5,60)	12(4.75,36)	-1.016	0.309
CRP	0.5(0.5,0.5)	0.5(0.5,0.625)	-0.966	0.334
ESR	14(10,23)	23(12.75,34)	-2.364	0.018*
PLR	114.44(77.26,165.34)	136.08(110.63,167.53)	-2.108	0.035*
SII	242.61(153.13,474.97)	292.57(191.11,460.31)	-1.363	0.173
NLR	1.46(1.21,2.31)	1.49(1.14,2.11)	-0.410	0.682
FAR	0.068(0.061,0.076)	0.071(0.062,0.081)	-0.723	0.470
G	31.8(26.58,34.9)	33.5(29.3,38.0)	-1.849	0.064
IgG	16.2(13.63,19.48)	17.85(14.88,22.55)	-1.927	0.054
ANA	320(160,320)	320(160,640)	-2.760	0.006*
anti-SSA/Ro52, n (%)	23(67.6)	111(75.5)	0.888	0.346
anti-SSA/Ro60, n (%)	27(79.4)	104(70.7)	1.037	0.309
anti-SSB/La, n (%)	11(32.4)	67(45.6)	1.969	0.161
ACA, n (%)	9(26.5)	35(23.5)	0.106	0.744
RF, n (%)	19(55.9)	83(56.5)	0.131	0.718
TMH	0.22(0.17,0.28)	0.21(0.16,0.27)	-0.872	0.383
av-NIKBUT	9.20(4.89,13.26)	6.48(4.18,10.40)	-2.128	0.033*
MGDS	2(1,3)	2(1,2)	-0.227	0.821

**Table 5:** Binary logistic regression analysis for positive labial gland pathology

	B	SE	Wald	*P*	OR	95%CI	
						Lower Limit	Upper Limit
ESR	0.021	0.015	1.850	0.174	1.021	0.991	1.051
PLR	0.009	0.004	4.327	0.038	1.009	1.001	1.018
ANA	0.002	0.001	2.959	0.085	1.002	1.000	1.004
av-NIKBUT	-0.056	0.031	3.129	0.077	0.946	0.889	1.006

**Table 6:** Stratified binary logistic regression analysis for positive labial gland pathology

	B	SE	Wald	*P*	OR	95%CI	
						Lower Limit	Upper Limit
ESR	0.023	0.016	2.162	0.141	1.023	0.992	1.055
PLR Grouping	1.045	0.411	6.469	0.011	2.842	1.271	6.358
ANA	0.002	0.001	2.910	0.088	1.002	1.000	1.004
av-NIKBUT	-0.054	0.032	2.911	0.088	0.948	0.891	1.008

## Discussion

PLR, SII, NLR, and FAR are established biomarkers of systemic inflammation, correlated with disease activity across various autoimmune diseases, and provide practical tools for clinical evaluation. In rheumatoid arthritis and psoriatic arthritis, elevated SII levels were linked to disease severity [6–9]. AFIFI N et al. observed higher FAR values during active phases of rheumatoid arthritis [10]. A study on systemic lupus erythematosus(SLE) defined clinical thresholds for NLR and PLR as indicators of disease activity [11]. Similarly, in SjD, UZELI Ü et al. found elevated SII, PLR, and NLR levels compared to healthy individuals, with SII correlating with ESSDAI scores [12]. MIHAI A et al. highlighted PLR’s predictive value for cutaneous vasculitis in SjD [13], and also found NLR could be helpful in predicting the neurological involvement in SjD patients [14]. However, the relationship between PLR and exocrine gland involvementremains underexplored in SjD. This study investigates PLR’s association with ocular surface damage and labial gland pathology in newly diagnosed SjD patients to evaluate its clinical application value.

Our study showed a positive correlation between PLR and labial gland pathology positivity. Multivariate analysis confirmed PLR as an independent predictor, with high-PLR patients at a 2.8-fold higher risk. PLR inversely correlated with TMH with a cutoff of 151.34 predicted TMH abnormality. These results suggest that PLR may serve as a marker of exocrine gland lymphocyte infiltration and dysfunction in SjD patients.

Multiple studies have confirmed that PLR is elevated in autoimmune diseases [15, 16] and the elevated PLR reflects thrombocytosis and/or lymphocytopenia. Platelets, beyond their role in hemostasis, significantly enhance inflammation by releasing cytokines and chemokines [17]. They also promote the formation of neutrophil extracellular traps (NETs), which, in turn, promote local platelet activation, exacerbating the immune response and sustaining chronic inflammation [18]. In SjD patients, peripheral lymphocytopenia may result from multiple mechanisms, including lymphocyte migration to target organs, B/T cell subset imbalance, and autoimmune-mediated lymphocyte destruction [19, 20]. Based on these findings, SjD patients with elevated PLR are more likely to exhibit inflammatory infiltration and tissue damage in salivary and lacrimal glands, as confirmed by our study. Thus, PLR, a simple and cost-effective blood marker, can serve as a valuable tool for assessing exocrine gland involvement and predicting labial gland pathology in SjD.

Our study found that PLR positively correlates with SII, NLR, and FAR, but showed no correlation with traditional inflammatory markers such as ESR and CRP. ESR is primarily influenced by plasma protein levels (particularly immunoglobulin), while CRP is an acute-phase protein produced by the liver in response to inflammatory cytokines such as IL-6. In contrast, PLR reflects the dynamic balance between platelets and lymphocytes. The characteristic of SjD is lymphocytic infiltration of exocrine glands, and PLR may capture this feature more effectively than ESR and CRP. Indeed, our study showed that labial gland pathology positivity correlates with PLR but not with ESR/CRP. We highlight the complementary role of PLR in SjD assessment, as it captures disease-specific features, particularly in predicting exocrine gland involvement.

Our study found no correlation between PLR and specific immune markers such as autoantibodies (ANA, anti-SSA, anti-SSB) or immunoglobulins (globulin, IgG). This highlights the complex pathogenesis of SjD, which involves a network of immune cells, particularly abnormal activation and dysfunction of B cells [21]. PLR reflects the total lymphocyte count but does not distinguish between lymphocyte subsets, such as B cells and T cells. Studies have shown that SjD patients with anti-SSA/SSB positivity exhibit distinct B-cell subpopulation abnormalities, such as increased naive B cells and decreased memory B cells [20]. Additionally, there were increased CD28^null^ T cells and dysfunctional Treg cells in SjD patients [22, 23]. These specific immune cell changes may drive the production of autoantibodys and immunoglobulins, yet they cannot be captured by PLR. This explains why PLR can predict labial gland pathology but shows no correlation with serum immune markers.

This study has several limitations. ​First, the single-center recruitment of participants may restrict the generalizability of our results. Second, the correlation between the ESSDAI score for SjD activity assessment and PLR was not included in the study. Future multi-center, large-sample studies will be conducted to further verify the predictive value of the model based on PLR.

In conclusion, PLR is a simple and accessible marker that can predict target-organ involvement, such as in salivary and lacrimal glands. It is best positioned as a complementary tool in SjD management.

## Data Availability

No datasets were generated or analysed during the current study.
